# One Step Forward: Development of a Program Promoting Active School Transportation

**DOI:** 10.2196/resprot.9505

**Published:** 2018-05-08

**Authors:** Anna-Karin Lindqvist, Stina Rutberg

**Affiliations:** ^1^ Department of Health Sciences Luleå University of Technology Luleå Sweden

**Keywords:** exercise, active commuting, health promotion, empowerment, gamification, child, school, social cognitive theory, intervention mapping

## Abstract

**Background:**

Physical activity promotes health and learning. However, up to 80% of the children in industrialized countries do not achieve the recommended level of daily physical activity. By encouraging children to use active school transportation (AST), it is possible to increase their overall physical activity.

**Objective:**

The aim of this paper was to present the development of an AST intervention using Intervention Mapping (IM) to promote children’s physical activity.

**Methods:**

The principles of IM were applied to guide the development of the intervention. The process was divided into 3 phases. First, a literature review and collection of experiences of stakeholders were carried out to gain a broad perspective on the problem and possible solutions. Thereafter, an analysis of the critical environmental and behavioral factors affecting outcome was conducted, which guided the choice of tangible components of the intervention. Finally, a plan of evaluation and implementation was established.

**Results:**

A structured program to increase AST among children was developed, consisting of 3 subsequent phases that are described in detail. Implementation took place, and evaluation of the intervention is being carried out.

**Conclusions:**

IM proved to be a valuable method to develop a structured AST intervention for children. By following the steps of the IM process, it became evident that empowerment and gamification are 2 promising avenues to consider when designing AST interventions in a school context. By engaging end users and including important agents, such as parents and teachers, who control the environmental factors, the possibility to design a sustainable program increases. In addition, gamification made it possible to integrate learning into AST, which could motivate schools to devote time and effort to implementing this program.

## Introduction

### Background

Although many studies have illuminated the ways in which physical activity provides children with fundamental health benefits, children are not physically active to the extent that is needed [1]. Among Swedish children aged 11 years, only 21% of males and 13% of females achieve the recommended daily levels of physical activity [2]. This is even more problematic when considering the increasing problem with childhood obesity, which has risen substantially in most high-income countries over the last three decades [3]. To reach the recommended levels of activity, children could be encouraged to walk or bike to school, which are known as forms of active school transportation (AST) [4]. However, many industrial countries have seen a decline in AST among children and adolescents, and an additional decline is likely in the absence of interventions to increase AST [5,6]. Though many studies have focused on the social and environmental factors that influence children’s AST, the interventions developed have proven insufficient for promoting AST [7]. Only a few schools implemented these interventions, perhaps as a result of the disconnect between research and schools’ needs [8]. To date, several concerns about school interventions for promoting physical activity have been highlighted in reviews. It has been pointed out that research has failed to determine the contextually sensitive attributes that define successful school-based interventions [9], and rigid scientific methodologies and evaluation techniques are incompatible with societally complex issues [10]. Given the disconnect between what was intended and how AST is carried out, there is a need to analyze physical activity interventions in schools to find ways to increase their feasibility and sustainability.

This paper aims to elucidate the logic model for the problem and the solution, as well as the theoretical underpinnings of an intervention promoting AST. Intervention research is defined as the process of creating the elements of an intervention and refining those elements through a series of studies [11]. By using theory and evidence as foundations in intervention research and by presenting multiple theoretical and experiential perspectives to solve a problem [12], other research teams can be inspired to build on this or prevent future mistakes. Intervention Mapping (IM) is both a framework and a structured way of planning, implementing, and evaluating health promotion programs [13]. The theoretical underpinning of IM is the social ecological model, which states that health is a function of the individuals living in an environment. Solving a health promotion problem requires a systematic perspective. The environments include physical, social, and cultural factors, in addition to agents exercising control over these factors at each ecological level, such as interpersonal, organizational, community, or societal environments.

### Objective

The aim of this paper was to present the development of an AST intervention using IM to promote children’s physical activity.

## Methods

### Intervention Mapping Tool

IM, a 6-step tool that maps the path from recognizing a problem to identifying a solution, was used to develop the program. The process was iterative rather than linear and the planners moved back and forth, as they gained new information and perspectives. The 6 steps include (1) logic model for the problem, (2) logic model for the solution, (3) program design, (4) program production, (5) program implementation plan, and (6) evaluation plan [13]. During program planning and execution, all planning group meetings were documented, as were field notes from every step of the process.

### Participants

Program development was driven by a planning group consisting of the 2 authors with research experience concerning children and physical activity, one researcher with research experience concerning AST with a special focus on environmental engineering, 2 teachers, the principal, and the head of the municipality’s planning department.

The research project was performed in a municipality of approximately 80,000 inhabitants situated in northern Sweden. Five schools with infrastructure that could allow first-grade children to walk or bike to school were invited to participate in the study, and one school agreed to participate. This primary school had 270 schoolchildren, and the neighborhood included both apartment houses and detached houses. The 2 cohorts consisted of 45 children aged 7 to 8 years (25 males). The parents and the children were informed about the study by the authors, both face-to-face and in writing, and 42 children (23 males and 19 females) agreed to participate. All parents were also invited to participate, and 63 parents (26 fathers and 37 mothers) did so. Of the 63 parents, 46 had a college/university education and the remaining 17 had an upper secondary education. All but 2 were Swedish citizens. The distance between home and school varied between 0.2 and 6.0 km, with an average distance of 1.3 km.

### Procedure

Program development began by forming the planning group, which was active during the whole project.

#### Phase 1

First, a logic model for the problem was formulated, which contained an assessment of the determinants and behavioral and environmental factors. On the basis of this model, a logic model for the solution was created. Gradually, these steps became increasingly intertwined. The phase was informed by a review of the theory and literature concerning children’s physical activity interventions (in particular AST, empowerment, and gamification). In addition, several other contacts were made within the municipality, surrounding community, and related scientific areas of research in order to understand the community, its members, and the theoretical models for the problem and the solution. We created a joint logic model for the problem and for the solution, which were inspired by the 2 models of Bartholomew Eldredge [13].

#### Phase 2

Once the theoretical framework was established, it was used to guide the tangible program components. To be true to an empowerment approach, the program development was a partnership between the teachers, parents, and children that created something that they perceived to be meaningful.

#### Phase 3

This phase included a plan for evaluation and implementation. Preliminary results were presented and discussed during this phase.

### Ethical Considerations

The study was performed in accordance with the principles of the Swedish law for research ethics and the Declaration of Helsinki’s Ethical Principles for Medical Research Involving Human Subjects [14]. The study was approved by the Regional Ethical Board in Umeå, Sweden, before the start of the research project (issue date: February 9, 2016; application registration number: 2015/296-31Ö).

## Results

The outcomes of the IM process are described in 3 different phases. The process began with the formulation of a logic model for the problem and the solution, and it continued with the program design and production. Ultimately, an evaluation plan was established.

### Phase 1: Logic Model for the Problem and Logic Model for the Solution

#### Theory- and Evidence-Based Factors Affecting Active School Transportation Behavior

To identify the factors related to AST behavior, social cognitive theory was used because it has been proven useful for developing effective physical activity behavior change interventions in children [15]. Social cognitive theory conceives of individuals within a collective context, as people do not operate as isolated individuals [16]. Social cognitive theory specifies a set of constructs, including knowledge, perceived self-efficacy, outcome expectations, goals, perceived facilitators, and impediments to change. Self-efficacy is altered by direct mastery experience, vicarious experience, and social persuasion [16]. Thus, self-efficacy might be applicable to this program because it could affect AST performance, modeling, and social support. Previous studies confirmed that self-efficacy mediates the causal pathway between interventions and children’s physical activity levels [17]. However, research examining social cognitive theories and physical activity has largely focused on adult populations, and there is limited knowledge regarding children [12].

Research has recognized that gamification has great potential to promote children’s physical activity and learning [18,19]. Gamification is defined as the use of game design elements in a nongame context [20]. The use of gamification enhances the possibility of capturing the components that make games engaging, and it can be used to improve the effectiveness of health promotion initiatives [21]. Promising research suggests that gamification can promote AST [22]. By using elements such as recurrent assignments that grant badges to students and allow them to level up to the next challenge, this approach can potentially ignite internal motivation to engage in healthy behavior. Acquiring badges can drive knowledge acquisition and behavior change [23].

The participatory elements of empowerment can improve an intervention’s compatibility, and they increase the likelihood that effective programs will be sustained [24]. One study has shown that problems with intervention design and evaluation can be overcome by taking advantage of the end users’ involvement and by explaining the connections among programs, policies, and evaluations [25]. We have previously performed promising school-based research using empowerment in order to promote physical activity [15]. According to Tengland [26], using an empowerment approach involves minimizing the influence of professionals, and the individual or group that is in need of support should take responsibility for the change process. Furthermore, empowerment and children’s active participation can increase knowledge acquisition and competencies [27]. Yet, critics have claimed that professionals should not reduce their power over projects because professionals are part of the project and must have a say in decisions [26].

#### Agents Who Control Environmental Factors

In the social ecological model, parents are an important interpersonal factor. Because children in this program were only 7 to8 years of age, their parents are the gatekeepers of their children’s AST. Our previous research concluded that parents are important as role models, providing encouragement and tangible support [15]. Therefore, parental attitudes toward AST are a factor that could constitute either a problem or a means of facilitation. Using parental attitudes as a starting point and including them in program development are consistent with Bandura’s theories [16] because environmental factors (such as social support) are a central element of both social cognitive theory and previous research [28]. However, few interventions have been based on parents’ psychological factors on an intrapersonal level, such as parents’ perceived barriers, outcome expectations, and self-efficacy [29].

An organization-level factor that is causally related to successfully increasing AST is school involvement. Promoting health might appear to be an added burden when the primary focus of schools is to meet academic standards. In fact, physical activity is sometimes seen as a competitor to academic studies because the time devoted to physical activity could instead be devoted to academic work [30]. Good health is critical for achieving an optimal education, and studies have found associations between children’s physical activity and academic performance [31]. Moreover, research has shown that physical activity improves children’s cognition and brain health [32]. This association should be a motivator for schools to be involved in increasing AST.

In this study, teachers were highly involved in the development of the program. For instance, they created special weekly assignments, were responsible for measuring distances for each child’s AST, and helped determine how to use these data in their teaching. Therefore, engaging teachers is required for the success of an AST program.

### Phase 2: Program Design and Production

Once the theoretical framework was established, the planning group addressed the critical environmental and behavioral factors that were most likely to affect the outcome (Textbox 1).

**Critical environmental and behavioral factors and actions that promote active school transportation (AST).**

- Empowerment of school personnel
  - Action: Planning workshops, assignments, and measurement of AST
- Knowledge and motivation of parents
  - Action: Parenting meeting: discussion, assess attitudes toward AST
- Knowledge of children
  - Action: Workshops on health, environment, and safety
- Empowerment of parents and children
  - Action: Bring the ideas of parents and children into the AST program
- Motivation of children
  - Action: Gamification of AST measurement and weekly assignments incorporated into teaching

Environmental factors we identified were parental knowledge of AST, attitudes toward AST, and perceived AST barriers. Therefore, during a parenting meeting, we informed parents about the benefits of physical activity for their children in terms of both health and academic performance. We also let parents discuss their doubts and fears around letting their children walk and bicycle to school and discussed solutions. In addition, we assessed parental psychological factors on an intrapersonal level using a questionnaire and discussions during this meeting. We analyzed the results from the questionnaire, determined their specific worries, and suggested solutions that were used as a starting point for program design. The questionnaire was the Modified Integrated model for Children’s Active Commuting to School, which has been shown to fit well with this model, and thus, may enable health behavior researchers to design effective interventions to promote AST [29]. Parents highlighted 2 main concerns: (1) having an accident when crossing roads with heavy traffic and (2) meeting a harmful person (stranger danger) while in transit during AST. Examples of suggested solutions used in the program were pairing children according to geographic location and encouraging them to walk or bicycle together to school. Children whose paths included heavily trafficked roads were accompanied by parents or older children.

Other behavioral factors we identified were the children’s knowledge of the benefits of AST and their motivation to engage in behavior change. To further improve the program, the next step involved collecting the children’s ideas on how to develop the program, as well as increasing their knowledge and motivation through workshops. The workshops had 3 different themes: (1) health and how physical activity affects the body and mind, (2) transportation safety (especially concerning bicycling and bullying), and (3) environmental effects of AST. The knowledge the children gained during the workshops was later used in the intervention period during standard school lessons. The workshops lasted for approximately 45 min, and information and discussions were mixed together. Examples of schoolchildren’s ideas on how to develop the program included encouraging pep talks within the pairs to increase AST use. Moreover, the children and their teachers worked collaboratively to divide the children into small groups or pairs according to where their homes were situated, constituting an empowerment approach.

To further enhance the children’s motivation to use AST, the program used gamification elements. Every day during the 4-week test period, the children put a sticker on a collective board for every kilometer they walked or bicycled between home and school, and the board’s results were integrated into lessons. For example, Mathematics class was integrated with Geography class, and the children summed the total number of kilometers achieved each week, converted kilometers into miles, and identified the locations on a map. In our previous research, we found that measuring physical activity was an effective part of a school-based intervention, and this motivated adolescents to be more physically active [33].

Another gamification element was special assignments (a challenge) that the children were encouraged to solve each week. The teachers selected the assignments, which were directly connected to the curriculum. One of the assignments on the first week was, “Which traffic signs do you encounter on your way to school?” The assignments were integrated throughout the curriculum. For example, in the Art class, the children painted pictures of traffic signs. Another assignment during the week was a security check of the bicycles in which the children followed a checklist concerning legal requirements for bicycle equipment. The second week’s special assignment was, “Count how many people you meet when walking or bicycling on your way to school,” and this assignment was integrated into the Math class. The assignment for the third week was “Bring a plastic bag and collect some litter on your way to school.” This assignment was used in a lesson concerning the environment in which the children learned about and practiced sorting litter into plastic, paper, etc. They also learned about what happens to animals and nature if the litter is left out in the environment. The fourth week’s assignment was “Notice which signs of spring you encounter on your way to school,” and this assignment was integrated into Biology class. Each Friday, the teachers summarized the assignment for the week, and a badge was awarded to the class for successful achievement.

### Phase 3: Program Evaluation Plan and Implementation Plan

Developing a plan for the adoption, implementation, and sustainability of the program in real-life contexts began when the needs assessment was undertaken, as careful examination of the end users’ needs contributed to program compatibility. To evaluate the program, we collected experiences from the children and teachers through focus groups. We used a semistructured interview guide to cover all aspects of the program. The opening question was “Let’s pretend I know nothing. Could you please tell me about the program?” To expand upon the answers, follow-up questions were asked. Seven focus groups with 4 to 5 children in each group and one focus group consisting of the teachers were convened. The focus groups’ discussions lasted for 16 to 45 min. Collectively, the 42 children walked or biked for 1189 km over 4 weeks, which averaged to 1.4 km for each child per school day. Bicycling contributed to at least 15 min of extra physical activity each day. The preliminary analysis of the focus groups showed that the students got motivated by the gamification elements of the intervention (ie, incorporating learning activities into assignments and measures of the use of AST), and they gained learning outcomes using time outside scheduled school hours. Furthermore, the teachers in this project found it highly rewarding to incorporate learning into AST because it made it possible to use real-life situations to teach various subjects in the curriculum.

In addition, before the intervention, we collected data on parental attitudes using the Modified Integrated model for Children’s Active Commuting to School questionnaire, and the parents answered an open letter as well, which was introduced with the following text: “You have answered a questionnaire concerning AST, including obstacles your child might experience while using AST. Describe how these obstacles can be overcome.” Two weeks after ending the intervention, a second open letter was introduced with 4 questions: “How have you as a parent experienced your child’s participation in the AST project”; “If this project is used in a different class, is there something we should do again, and are there things that should be changed”; “Which attitude towards AST did you have before the intervention, and have your attitudes changed after participating”; and “Has your own choice of travel modes changed during the project?”

The knowledge gained from these data will provide important information on key program components, which will enable us to develop sustainable health-promoting programs in a school context. We have planned to implement this program in several schools, and after implementation, feasibility and efficacy studies using cluster randomization are planned.

## Discussion

### Principal Findings

In this paper, we describe the systematic development of an intervention program aimed at promoting AST among children. By following the steps of the IM process, it became evident that empowerment and gamification are 2 promising avenues to consider when designing AST interventions in a school context.

Using empowerment and engaging end users was essential. By forming a planning group that included important agents who control the environmental factors, it was possible to create a promising approach for developing a sustainable physical activity program in a school that could yield positive outcomes for children. In young children, parents are the gatekeepers and they decide whether the child will use AST or other means of transportation. Thus, it is essential to involve them in the process. However, this approach is unusual. A common theme that occurs in many school-based research articles is the lack of engagement by end users in planning, implementing, and evaluating health promotion activities [25,34]. Therefore, we anticipate that one success contributor for this program was the use of empowerment.

A second important cornerstone was the use of gamification, which made it possible to integrate learning and AST through the use of school assignments, including ones that incorporated measurements of AST usage. Preliminary finding showed that gamification motivated children to use AST, a finding that is supported by Hamari et al [35]. There is an ongoing debate within gamification research as to whether specific game elements may actually undermine users’ intrinsic motivations [36]. A study examining the effects of 3 commonly employed game design elements (points, leaderboard, and levels) on users’ performance and intrinsic motivation showed that these game elements significantly increased performance but did not affect intrinsic motivation. These findings suggest that points, levels, and leaderboards by themselves do not make or break users’ intrinsic motivation in nongame contexts [36]. However, the use of gamification in schools must be further explored, as the relationship between the engaging aspect of games, learning, and physical activity is still unknown [37].

### Future Work

By integrating learning activities into the project, schools may be more motivated to put time and effort into implementing this program. There are complex interactions among socioeconomic, environmental, and ethnic and cultural differences, and these may be important to account for when designing effective programs to promote children’s AST. Future evaluation methods need to include a target group that displays a wider range of socioeconomic factors.

### Conclusions

IM proved to be a valuable method to develop a structured intervention for an active school transporting intervention for children. By following the steps of the IM process, it became evident that empowerment and gamification are 2 promising avenues to consider when designing AST interventions in a school context. By engaging end users and including important agents, such as parents and teachers, who control the environmental factors, the possibility to design a sustainable program increases. In addition, gamification made it possible to integrate learning into AST, which could motivate schools to devote time and effort to implementing this program.

## Abbreviations

AST: active school transportation
IM: Intervention Mapping
